# Socioeconomic and Demographic Factors Affecting Body Mass Index of Adolescents Students Aged 10–19 in Ambo (a Rural Town) in Ethiopia

**Published:** 2010-12

**Authors:** Mesert Yetubie, Jemal Haidar, Hailu Kassa, Fleming Fallon

**Affiliations:** 1 *Oromia Health Bureau, Ethiopia;*; 2 *School of Public Health, Addis Ababa University, Ethiopia;*; 3 *Bowling Green State University, Bowling Green, Ohio, USA;*; 4 *Bowling Green State University, Bowling Green, Ohio, USA*

**Keywords:** sociodemographic factors, underweight and Ambo

## Abstract

**Background:**

Body mass index (BMI) is a commonly used anthropometric measurement to estimate the level of nutritional indices (underweight/overweight) of adolescents and adults. Knowledge of the factors affecting BMI is essential for developing intervention programs. This study was conducted to measure BMI and determine the socioeconomic and demographic factors affecting the relative weight of adolescents living in rural districts in Ethiopia.

**Methods:**

A randomized cross-sectional study of 425 adolescent students living in the Ambo region of Ethiopia was conducted. A pre-tested questionnaire was used to collect the socioeconomic and demographic status of the participants. BMI (weight/height^2^, kg/m^2^) was measured and used as index of relative weight. Data were analyzed using SPSS version 15. A stepwise backward logistic regression analysis was applied to identify the major determinant abnormal weight of the adolescents while controlling for co-linearity.

**Results:**

The prevalence of underweight was 27.2% with male preponderance. The proportion of normal weight females was higher than that of males. There was no significant difference in the proportion of overweight males and females. Age, number of meals, parental education level, source of food, and number of cattle owned were correlated with being underweight. After adjusting for confounding variables only being a member of the younger age group (AOR=1.99; 95% CI=1.01 to 3.57), source of food (AOR=2.4; 95% CI=1.24 to 4.74), and a greater number of cattle owned (AOR=4.9; 95% CI=1.27 to 19.28 were positively correlated with being underweight.

**Conclusion:**

Younger age adolescents, those who come from homes with no or few cattle, and their parents purchased food were likely to be underweight. This study will help local governments, educators and community groups develop programs to assist underweight adolescents attending schools.

## INTRODUCTION

Adolescents comprise one-fifth to one-quarter of the world’s population (1–2). Adolescence being an important physical growth period (3–5), it is affected by inadequate or inappropriate food intake (3–8) due to household economic and social disadvantages (3–5).

In many developing countries, nutrition initiatives have focused on children and women, resulting in adolescents often been neglected (4–7). This has caused adolescent to suffer from many health problems later in life (4). Demographic factors such as age, gender, have been linked to adolescent nutritional status. Previous studies from developing countries have indicated that younger adolescents are at greater risk of being undernourished than their older counterparts, with the risk increasing in adolescents living in rural than in urban areas (4–6). A recent study conducted in china found that the BMI of adolescents living in rural areas was significantly lower than those living in urban areas (9). In many developing countries, the rate of undernourishment among boys is almost twice that of girls (4).

In Ethiopia, undernourishment among preschool aged children has been well documented (10), but studies on factors affecting the nutritional status of adolescents have not been studied in detail (11). The available studies focused on factors affecting pregnancy outcomes rather than on problems associated with normal growth and development of adolescents (10, 11). This study was conducted to investigate the socioeconomic, demographic and other important characteristics affecting BMI of adolescents aged 10−19 years attending schools in a rural community in Ethiopia. The information obtained can be used to improve existing policies and programs targeting adolescent nutrition.

## METHODS

### Study Area

The study was conducted in Ambo, located 105 kilometers west of Addis Ababa. It is representative of a typical small rural community in Ethiopia with some level of infrastructure development, such as paved roads, electricity, communication system, small scale factories. The population of Ambo is 41, 133, has a male to female ratio of 0.98:1 and is ethnically mixed (12). It has a secondary school and four elementary schools. Like in other rural communities in Ethiopia, adolescents attending schools in this community come from different socioeconomic backgrounds.

### Study Subjects

Students from the secondary school and from one of the four elementary schools were recruited for the study. Because of logistical constraints, the number of participating elementary schools was restricted to one and randomly selected by a lottery method. Out of a total of 2930 adolescents (n=1470 in primary schools and n=1460 in secondary schools and institutes), 425 subjects (n=213 in primary schools and n=212 in secondary schools) were selected randomly for the study using the same lottery method. The sample size was determined using the following parameters: an assumed underweight rate of 50%, a non-response rate of 10%, a 95% confidence level and a precision rate of 5%. Data were collected between March 20 and April 10, 2008.

The study was reviewed and approved by the Research and Ethical Clearance Committee of the Medical Faculty of Addis Ababa University, an institutional review board. After full explanation of the purpose and nature of the study to the eligible, verbal consent was obtained from the eligible as well as parents or guardians of adolescents selected for the study.

### Study instruments

The instruments used in this study were adapted to the socio-cultural setting of the region through expert opinion. Before the actual study took place, the questionnaire to collect demographic, health and dietary data was prepared in English and pretested in the same region where the training was conducted. After making some corrections to the original instruments, the final version of the questionnaire was translated into the main local languages spoken in the region, namely Amharic and Oromiffa.

### Data Collectors

Two supervisors (one male and one female, teachers) and four data collectors (from the school community) were recruited and trained for 3 days by the principal investigator. To maintain consistency in the process of data collection, the anthropometric measurements of the subjects were taken by the two supervisors after their methods of measuring were standardized.

Data collectors interviewed the respondents for their socio-demographic profile in a private room. The respondents were parents or family members related to the study subjects. A gross clinical assessment for signs and symptoms of common nutritional deficiencies was done by the principal investigator. The principal investigator followed protocols to standardize data collection methods.

### Anthropometric Measurement

The nutritional index of adolescents was computed using body mass index (BMI = Kg/m^2^) as recommended by the World Health Organization. A digital bathroom scale was used to obtain weight. Prior to each weighing, the scale was adjusted to zero reading to enhance validity. Each participant was weighed twice to improve the accuracy and reliability of measurement; the mean value (to the nearest 0.1 kg, as recommended by the World Health Organization (13) was recorded. A standardized measuring board with a fixed head rest and a movable foot piece was used to determine height (13–14). Height was measured without shoes following standard procedures with the head in upright position and the body firmly stretched and resting on the board.

The following definitions were used to compute the nutritional indices of study participants: BMI for age value less than 5^th^ percentile as underweight, BMI for age-value greater than or equal to 5^th^ percentile but less than 85^th^ percentile as normal weight, BMI for age-value greater than or equal to 85^th^ percentile but less than 95^th^ percentile as overweight, and BMI for age-value greater than or equal to 95^th^ percentile as obese (14–15).

### Statistical analysis

Data were analyzed using SPSS version 15. Statistical calculations included descriptive statistics, bivariate analysis for associations between different variables, odds ratios with 95% confidence intervals for the degree of association between variables for identifying important determinants of adolescent relative weight. Then, a stepwise backward logistic regression analysis was applied to test further the observed significant variables in bi-variate analysis while controlling for co-linearity. A *p*-value below 0.05 denoted significance in differences.

## RESULTS

The demographic characteristics of the study participants are shown in Table 1. Of the 425 adolescents who participated in the study, over half (54.4%) were 15–19 years old. The mean age of study participants was 14.4 ± 3.2 years. The proportion of boys (56.9%) was higher than females (43.1%). The majority (94.8%) came from two-parent homes with other siblings living in the home. Over three quarter (76.2%) of the head of house-hold had education ranging from primary school to college level. The occupation of the father’s included working on self-owned farm (33.4%) government employee (33.6%), and the rest worked as day laborers or were self-employed. Most (43.5%) of the mothers were homemakers. The annual household income for a majority (58.9%) of the families was under $628 and more than half (54.8%) had no cattle.

**Table 1:** Demographic Characteristics of participants and their families

Characteristic	Number	Percent

Age (years):		
10–14	194	45.6%
15–19	231	54.4%
Gender:		
Female	183	43.1%
Male	242	56.9%
Marital status		
married	403	94.8%
single	22	5.2%
Family size:		
<4 members	70	16.5%
≥4 members	355	83.5%
Education status of family-head:		
No education	101	23.8%
primary	128	30.1%
secondary	127	29.9%
college	69	16.2%
Occupation (father):		
farming	142	33.4%
self-employed	78	18.4%
day laborer	43	10.1%
government	143	33.6%
other	19	4.5%
Occupation (mother):		
government	61	14.4%
self-employed	58	13.6%
farming	110	25.9%
homemaker	185	43.5%
other	11	2.6%
Annual family income in US$^*^		
<376	133	31.4%
377–627	117	27.5%
628–1,254	92	21.6%
≥1,255	83	19.5%
Cattle owned		
none	123	28.9%
1	17	4.0%
2	27	6.4%
3	111	26.1%
≥4	147	34.6%

At the time of the study the exchange rate was 1US$=9.57 Ethiopian Birr.

Of the participants, 68.5% had BMI in the normal weight category, 27.2% in the underweight category, and 4.4% in the overweight category (54.9%). When data was analyzed by gender, the proportion of underweight males was significantly higher (29.8%) than that of females in the same weight category (24.6%) (t=3.16; df=416; *p*<0.05). In contrast, the proportion of males (66.4%) in the normal weight category was significantly lower than that of females (70.5%) in the same weight category (t=5.31; df=423; *p*<0.05). The proportion of overweight females and males was 4.9% and 3.8%, respectively, but were not statistically different from each other (t=1.12; df=423; *p*>0.05).

When data was analyzed by age group, the proportion of 10-14 year olds in underweight category was significantly higher than the proportion of 15–19 year olds in the same weight category (t=20.02; df=423; *p*<0.05). In contrast, the proportion of 10–14 year olds in the normal weight category was significantly lower than the proportion of 15–19 year olds in the same weight category (*p*<0.05). Less than 5% of the adolescents were overweight and there was no significant difference between the age groups. (t=1.80; df=423; *p*>0.05) in this weight category (Table 2).

**Table 2:** Body weight category by gender and age as determined by Body Mass Index

Category	Gender		Age	
	Male	Female	10−14 years	15−19 years

Underweight	29.8%^a^	24.6%	38.1%^b^	18.6%
Normal	66.4%^a^	70.5%	58.7%^b^	76.2%
Overweight	3.8%	4.9%	3.1%	5.2%

a Difference between males and females is significant (*p*<0.05);

b Difference between younger and older adolescents is significant (*p*<0.05).

Eating 2 or less meals per day, parents having primary or less education, parents purchased, and parents owned 2 or less cattle and late menarche (15–19 years) were significantly associated with underweight (*p*<0.05). However, when adjusted for the confounding effect of other variables, underweight remained statistically significant among adolescents whose parents owned 2 or fewer cattle, ate purchased food, or consumed both purchased and home-grown food (Table 3).

**Table 3:** Bivariate and logistic regression analysis of socioeconomic factors associated with adolescent underweight

Variables	Underweight	COR (95% CI)	AOR (95% CI)

Age			
10 to 14	74 (63.2%)	2.7 (1.73−4.18)^b^	1.99 (1.01−3.57)^b^
15 to 19	43 (36.7%)	1	1
Meals/day			
≤ 2 meals	31 (26.5%)	0.4 (0.20−0.85)^b^	0.6 (0.09−4.12)
3 meals	72 (61.5%)	0.7 (0.43−1.20)	0.9 (0.41−2.31)
≥ 4 meals	14 (12.0%)	1	1
HH-Head Education			
No education	30 (25.6%)	0.4 (0.23−0.89)^b^	0.7 (0.20−2.97)
Primary	35 (29.9%)	0.4 (0.26−0.93)^b^	0.7 (0.15−1.36)
Secondary	36 (30.8%)	0.6 (0.33−1.14)	0.6 (0.23−1.65)
College	16 (13.7%)	1	1
Food Source			
Farm (produced)	21 (17.9%)	1	1
Market (Purchased)	78 (66.7%)	2.3 (1.40−3.87)^b^	1.1 (0.22−6.08)
Both^a^	18 (15.4%)	3.4 (1.99−6.10)^b^	2.4 (1.24−4.74)^b^
Cattle Owned			
0	68 (58.1%)	3.4 (1.99−6.10)^b^	2.4 (1.24−4.74)^b^
1	13 (11.1%)	6.3 (1.68−23.91)^b^	4.9 (1.27−19.28)^b^
2	10 (8.5%)	4.4 (1.51−13.02)^b^	3.6 (1.23−11.09)^b^
3	12 (10.3%)	2.4 (0.78−7.59)	2.0 (0.64−6.49)
≥ 4	14 (12.0%)	1	1
Age of Menarche			
15 to 19 years	10 (22.2%)	2.3 (0.69−8.20)	1.9 (0.51−7.58)
10 to 14 years	4 (8.9%)	1	1

HH, Household; COR, Crude odds ratio; AOR, Adjusted odds ratio;

a Means produced little;

b Difference is statistically significant (*p*<0.05).

## DISCUSSION

In a study conducted in 2007 in Addis Ababa (capital city of Ethiopia), the prevalence of underweight among school age adolescent was 13.0% (17) which is significantly lower than that found in this study (27.2%). The difference in the underweight prevalence maybe attributed to income difference in the two locations. Addis Ababa being more urban than Ambo is, the adolescents in Addis Ababa probably had more access to food than were the adolescents in Ambo. Underweight rates among the adolescents in this study were similar to those found in rural Benin (18), India (19), and Nepal (20).

The widespread practice of vegetarianism in rural areas is thought to be responsible for low body weights in Asia (21). In Bangladesh, underweight adolescent girls with diet poor in protein and vitamins were found to be more likely to be underweight (22). In rural Ethiopia, most people obtain their energy from grain and consume little or no protein on a daily bases. More than 3 out of 5 (60.9%) of the study respondents reported consuming *teff*, a type of grain common in the Ethiopia cuisine, on a daily bases. It is plausible to assume that poor dietary habits often accompanied by lack of adequate amounts of proteins and vitamins contributed to being underweight among the adolescents in this study.

Adolescents from families that do not have cattle were likely to be underweight. Cattle are important sources of milk and meat. Families that are unable to raise their own cattle must purchase meat and milk from sources outside of the home (23). With money unavailable for such purchases, this approach is not likely to be successful. Without money a more likely outcome, considering the low per capita income in the region is substitutions of *teff* for milk or meat. The result is likely to be inadequate nutrition and underweight among adolescents. In many rural settings of Ethiopia, residents sell some portion of their milk and purchase other types of food for household consumption, resulting in low frequency of milk consumption. Poor dietary of milk is among the risk factors for the prevalence of underweight adolescent girls in Bangladesh and Nepal (22, 24).

The proportion of overweight girls was higher than the proportion of overweight males, but was not statistically significant. This is consistent with the number of meals that are eaten per day as well as the fact that boys consumed fewer helpings of meat, vegetables and fruit than females did. In rural Ethiopia, female’s adolescents did the household chorus such as cooking giving them more access to food. This finding is consistent with a finding of a study conducted in India (19). Age, in particular being younger, emerged as a risk factor for being underweight. Because older siblings are better able to compete for relatively scarce food, younger children may receive inadequate nutrition.

BMI is one of the factors influencing the age of menarche. Obese and overweight adolescent girls are more likely to attain menarche in early adolescent life than their counterparts who are underweight (25–27). Gradual reductions in the age of menarche have been associated with progressive improvements in education, economic status and nutrition in western societies (28). The median age of menarche among the adolescents in this study was 15 years, with the majority falling in the underweight category. This study showed that, girls who experience menarche at age 15 or older are more likely to be underweight than those experiencing menarche in early adolescent life (t=4.78; df=1; *p*<0.05). Comparable result was obtained in study conducted in western Kenya (29). Better nutrition tends to lower the age of menarche.

In conclusion, adolescent boys were more likely to be underweight than were adolescent girls. Boys ate fewer meals each day and were more likely to eat *teff* than meat, vegetables or fruit. Girls consumed significantly more portions of meat, vegetables and fruit. Younger adolescents are more likely to be underweight than were older adolescents. In addition, few cattle ownership, buying than producing food, and late onset of menarche were also associated with being underweight. Since, the underweight adolescents in this study come from poor income families, had few source of income for food, government and non-governmental agencies need to target the identified groups.

One of the limitations of this study may have been restricting the primary grade sample cohort to a single elementary school. If the students at the chosen school differed from those in the non-selected schools, this limitation would have validity. If the selected students did not differ from those not selected, the sample would be adequate. Based on personal knowledge of the schools and their students, we feel that the students from the selected school comprise a representative sample. Being careful, the results should be generalized to all primary schools in Ambo with caution. Secondly, the fact that the findings were based on anthropometric and clinical examination and were not accompanied by biochemical tests may have introduced some bias into the data.

## COMPETING INTERESTS

The authors declare that no conflicting interests exist.
